# Fundamental kinematic indicators predict surgical expertise and clinical outcomes in robot-assisted surgery

**DOI:** 10.1038/s41746-026-03137-9

**Published:** 2026-09-29

**Authors:** Hideto Ueki, Takuto Hara, Tomoko Yamaguchi, Naoto Wakita, Yasuyoshi Okamura, Kotaro Suzuki, Yukari Bando, Kiyoyuki Chinzei, Munenori Uemura, Tomoaki Terakawa, Akihisa Yao, Koji Chiba, Jun Teishima, Hideaki Miyake

**Affiliations:** 1 Department of Urology, Kobe University Graduate School of Medicine, Kobe, Japan; 2 Department of Medical Device Engineering, Kobe University Graduate School of Medicine, Kobe, Japan; 3 Department of International Clinical Cancer Research and Promotion, Kobe University Graduate School of Medicine, Kobe, Japan; 4 Faculty of Advanced Science and Technology, Kumamoto University, Kumamoto, Japan

**Keywords:** Biomarkers, Computational biology and bioinformatics, Health care, Medical research

## Abstract

The global proliferation of robotic surgical platforms necessitates a framework for objective skill assessment using fundamental kinematic features that are theoretically extractable from any robotic system with 3D position tracking. Here, we demonstrated that fundamental three-dimensional movement patterns alone can accurately distinguish surgical expertise and show associations with clinical outcomes. A Random Forest classifier discriminated expertise with high case-wise accuracy (area under the curve 0.892 ± 0.075), and feature-importance analysis identified left-hand peak density as the principal discriminator. A small set of these fundamental indicators generalized to unseen surgeons in leave-one-surgeon-out validation, localizing the expertise-associated signal to a few interpretable kinematic measures. Kinematic patterns were correlated with clinical outcomes, and exploratory analysis revealed associations between movement features and blood loss. Individual learning curve analysis revealed heterogeneous adaptation patterns among experts transitioning to the new platform and identified distinct phenotypes with implications for personalized training. These findings show that fundamental kinematic analysis using only three-dimensional positional data can provide interpretable indicators of surgical performance that may extend across robotic systems providing coordinate data.

## Introduction

The integration of artificial intelligence with robotic surgery offers unprecedented opportunities for objective performance assessment through the automated capture of high-fidelity movement data^1^. Comprehensive platform-specific metrics from established systems, such as the da Vinci, have exhibited strong correlations with clinical outcomes^2–4^; however, this approach faces fundamental limitations as diverse robotic platforms have emerged globally^5–15^. Each system’s proprietary data architecture presents barriers to standardized evaluation; yet, all systems must capture basic end-effector positions for control purposes.

According to motor learning theory, surgical expertise is expressed through fundamental movement patterns that are independent of the specific interface used. Evidence from laparoscopic surgery shows that simple metrics, such as path length, movement smoothness, and completion time, are strongly correlated with experience and outcomes^16^. This raises a critical question: Can positional data alone offer sufficient information for accurate skill assessment while enabling cross-platform generalizability?

The Hinotori Surgical Robot System, Japan’s first nationally developed platform, offers an ideal test bed for this hypothesis^17–19^. Its system architecture allows for the extraction of three-dimensional coordinate data, facilitating the investigation of movement-based assessment methods. In this study, using the Hinotori surgical robot system as an initial platform, we investigated whether kinematic signatures captured solely through position data could discriminate expertise levels and predict clinical outcomes, thereby exploring the potential for a generalizable framework applicable across robotic platforms that provide access to coordinate data.

## Results

### Cohort characteristics and surgical expertise distribution

The analysis involved 98 robot-assisted radical prostatectomy procedures with complete kinematic data capture during vesicourethral anastomosis. Sixteen surgeons performed the cases (range: 1–18, median: 6, interquartile range (IQR) 2–14), with certified proctors conducting 68 procedures (69.4%) and non-proctors 30 procedures (30.6%). Patient demographics did not differ significantly between the groups (Table 1), supporting that outcome variations are attributable to surgical technique rather than case-selection bias.

**Table 1:** Patient demographics and clinical characteristics show balanced groups

Variable	Proctor (*n* = 68)	Non-Proctor (*n* = 30)	*p*-value
Age (years)	68.1 ± 6.1	68.9 ± 6.9	0.393
BMI (kg/m^2^)	24.0 ± 2.7	24.4 ± 3.3	0.876
ASA-PS, *n* (%)			0.72
ASA-PS 1	7 (10.3%)	2 (6.7%)	
ASA-PS 2	57 (83.8%)	27 (90.0%)	
ASA-PS 3	4 (5.9%)	1 (3.3%)	
Initial PSA (ng/mL)	7.7 (5.4–10.6)	7.5 (5.6–10.0)	0.512
Prostate volume (mL)	36.0 ± 18.2	32.0 ± 14.6	0.217
Clinical T stage, *n* (%)			0.11
T1c	5 (7.4%)	0 (0.0%)	
T2	55 (80.9%)	29 (96.7%)	
T3	8 (11.8%)	1 (3.3%)	
Biopsy Gleason sum	7 (6–9)	7 (6–9)	0.46
D’Amico risk group, *n* (%)			0.117
Low	2 (2.9%)	3 (10.0%)	
Intermediate	38 (55.9%)	20 (66.7%)	
High	28 (41.2%)	7 (23.3%)	
Neoadjuvant hormone therapy	4 (5.9%)	0 (0.0%)	0.309
Surgeon experience (years)	24.6 ± 4.1	22.3 ± 6.6	0.12
Previous abdominal surgery	19 (27.9%)	5 (16.7%)	0.311

No significant differences were observed between procedures performed by proctor and non-proctor surgeons in terms of age, body mass index, American Society of Anesthesiologists physical status, initial prostate-specific antigen levels, prostate volume, Gleason scores, or clinical staging. These findings confirm that outcome differences reflect surgical technique rather than case complexity. Values presented as mean ± standard deviation, median (interquartile range), or n (%). Statistical comparisons were conducted using the Mann–Whitney U test for continuous variables and the chi-square test for categorical variables.

*BMI* body mass index, *ASA-PS* American Society of Anesthesiologists physical status classification, *PSA* prostate-specific antigen, *T stage* clinical tumor stage, *D’Amico risk* D’Amico risk classification.

### Movement quality as an indicator of surgical expertise

**Fig. 1:**
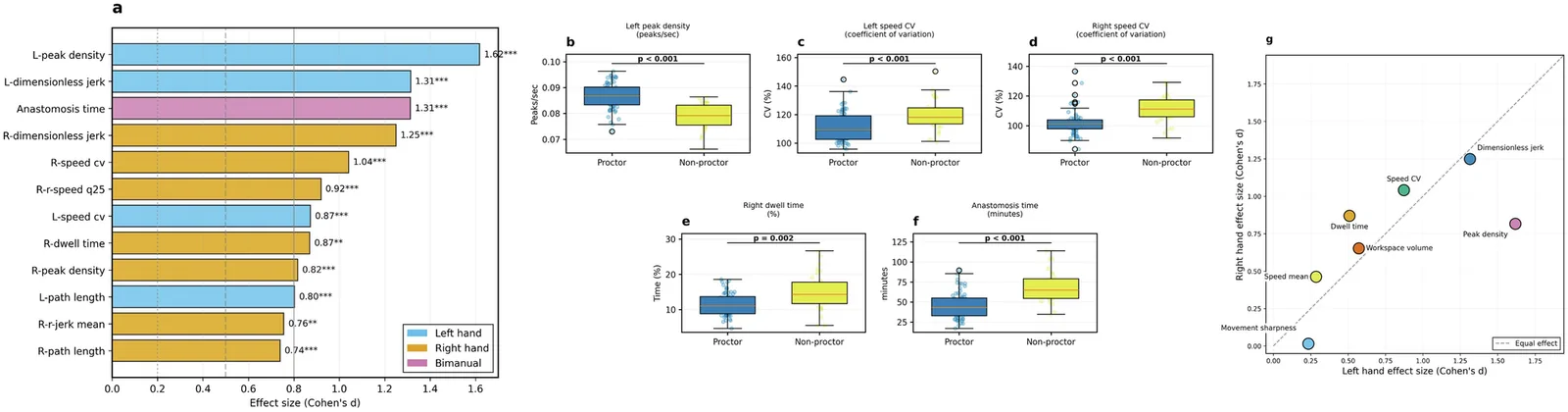
Kinematic features distinguish proctor from non-proctor surgeons. **a** Forest plot showing effect sizes (Cohen’s d) for features with the largest between-group differences. Features are color-coded: left hand (blue), right hand (orange), bimanual coordination (reddish purple). Significance levels are indicated using asterisks (*p < 0.05; **p < 0.01; ***p < 0.001; Mann–Whitney *U* test, false discovery rate corrected). Reference lines indicate small (0.2), medium (0.5), and large (0.8) effects. **b**–**f** Representative features illustrating group differences. Boxes show interquartile ranges with medians; whiskers extend to 1.5 times the interquartile range. Sample sizes: proctor *n* = 68, non-proctor *n* = 30. **b** Left peak density (peaks/sec). **c** Left speed coefficient of variation. **d** Right speed coefficient of variation. **e** Right dwell time percentage. **f** Anastomosis time (min). **g** Paired feature effect sizes for left versus right hand. Points above the diagonal indicate stronger right-hand discrimination.

The left peak density emerged as the most powerful discriminator of expertise (Fig. 1a, b).

Proctor surgeons demonstrated significantly higher movement consistency, with a left peak density of 0.087 ± 0.005 compared with 0.079 ± 0.005 in non-proctors (d = 1.618, *p* < 0.001), representing a 10.1% higher frequency of velocity peaks (Table 2, Supplementary Data 1). Movement smoothness metrics also exhibited strong discrimination: dimensionless jerk for left hand [(4.3 ± 2.3) × 10^5^ vs (7.8 ± 3.3) × 10^5^ (d = 1.314, *p* < 0.001)] and right hand [(4.2 ± 2.2) × 10^5^ vs (7.3 ± 3.0) × 10^5^ (d = 1.249, *p* < 0.001)], indicating 44% smoother movements in proctor surgeons. Temporal efficiency demonstrated a comparable effect size, with anastomosis times of 45.5 ± 16.1 min for proctors versus 67.8 ± 18.8 min for non-proctors (d = 1.313, *p* < 0.001), reflecting a 32.9% reduction. Fifteen of the 25 features (60%) achieved statistical significance after false discovery rate correction (Table 2, Supplementary Data 1). Notably, speed variability metrics showed strong bilateral discrimination, with a right speed coefficient of variation (*d* = 1.041) and left speed coefficient of variation (*d* = 0.873), both *p* < 0.001 (Fig. 1a–e). Left- and right-hand features exhibited differential effect sizes across feature categories: left-hand features showed stronger discrimination for movement-pattern metrics (peak density and movement sharpness) and path length, whereas right-hand features showed stronger discrimination for speed-related metrics and dwell time (Fig. 1g, Table 2).

**Table 2:** Group comparison of kinematic features between proctor and non-proctor surgeons

Feature	Proctor (*n* = 68)	Non-Proctor (n = 30)	Cohen’s *d*	*p* value	FDR-adjusted *p*
Movement Smoothness					
Left Dimensionless Jerk	(4.3 ± 2.3) × 10^5	(7.8 ± 3.3) × 10^5	1.314‡	<0.001	<0.001***
Right Dimensionless Jerk	(4.2 ± 2.2) × 10^5	(7.3 ± 3.0) × 10^5	1.249‡	<0.001	<0.001***
Left Jerk Mean	46.64 ± 11.61	41.58 ± 6.73	0.488	0.079	0.099
Right Jerk Mean	50.55 ± 10.24	43.32 ± 7.83	0.756	0.004	0.007**
Speed Characteristics					
Left Speed Mean	16.45 ± 4.01	15.42 ± 2.43	0.285	0.576	0.6
Right Speed Mean	17.90 ± 3.59	16.36 ± 2.60	0.462	0.14	0.167
Left Speed Cv	1.11 ± 0.10	1.20 ± 0.10	0.873†	<0.001	<0.001***
Right Speed Cv	1.02 ± 0.08	1.11 ± 0.09	1.041†	<0.001	<0.001***
Left Speed Q25	3.41 ± 1.43	2.68 ± 0.86	0.568	0.041	0.06
Right Speed Q25	5.21 ± 1.16	4.10 ± 1.31	0.919†	<0.001	<0.001***
Movement Pattern					
Left Peak Density	0.087 ± 0.005	0.079 ± 0.005	1.618‡	<0.001	<0.001***
Right Peak Density	0.082 ± 0.004	0.078 ± 0.004	0.817†	<0.001	<0.001***
Left Movement Sharpness	47.3 ± 9.3	49.3 ± 6.7	0.234	0.078	0.099
Right Movement Sharpness	48.1 ± 9.4	48.2 ± 6.1	0.016	0.269	0.305
Left Dwell Time	18.2 ± 6.4	21.3 ± 5.3	0.509	0.036	0.057
Right Dwell Time	11.5 ± 3.1	14.9 ± 5.2	0.870†	0.002	0.004**
Workspace Utilization					
Left Workspace Volume	16857.7 ± 8891.8	22204.3 ± 10375.6	0.571	0.009	0.017*
Right Workspace Volume	17557.1 ± 7298.8	22787.8 ± 9475.5	0.652	0.012	0.019*
Path Characteristics					
Left Path Length	45390 ± 22185	62918 ± 21023	0.803†	<0.001	<0.001***
Right Path Length	49666 ± 23273	66204 ± 20110	0.739	<0.001	<0.001***
Bimanual Coordination					
Speed Correlation	0.50 ± 0.07	0.55 ± 0.07	0.688	0.003	0.007**
Hands Distance Mean	246.02 ± 52.57	249.86 ± 49.45	0.074	0.652	0.652
Motion Synchrony	42.7 ± 6.6	46.4 ± 7.5	0.534	0.064	0.089
Bilateral Still Sync	4.7 ± 2.0	4.4 ± 2.1	0.159	0.421	0.457
Temporal					
Anastomosis time (min)	45.5 ± 16.1	67.8 ± 18.8	1.313‡	<0.001	<0.001***

Data are presented as mean ± SD.

*p* values were calculated using Mann–Whitney *U*-test.

FDR correction was performed using Benjamini–Hochberg method.

Cohen’s *d*: †Large effect (|*d*| ≥ 0.8), ‡Very large effect (|*d*| ≥ 1.2).

Significance levels: *p < 0.05, **p < 0.01, ***p < 0.001 (FDR-adjusted).

Features are grouped by category with effect sizes (Cohen’s *d*), *p* values, and false discovery rate-adjusted *p* values. Movement-pattern consistency, led by left peak density, showed the largest discriminative power. Effect sizes are interpreted as follows: small (0.2), medium (0.5), large (0.8), very large (>1.2). Workspace volume is a bounding-box measure and is sensitive to spatial outliers. Under a 5th to 95th percentile robust estimate, the between-group difference attenuated and lost significance for the left hand (Cohen’s *d* 0.57–0.29) and was reduced for the right hand (*d* 0.65–0.52). See Limitations.

*FDR* false discovery rate.

### Machine learning validates objective expertise classification

**Fig. 2:**
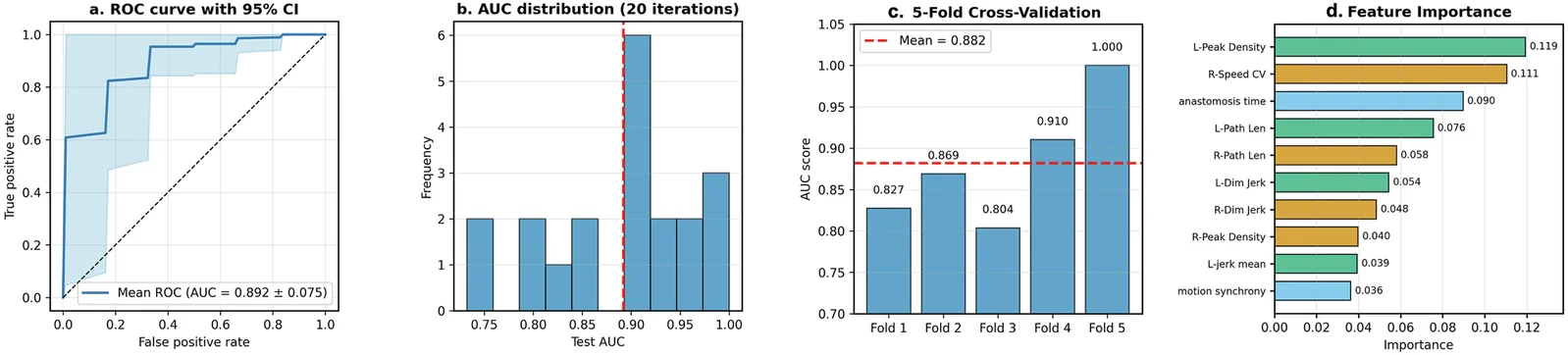
Machine learning classification of surgical expertise. **a** Receiver operating characteristic curve for Random Forest classifier. The thick line represents mean performance across 20 iterations; the shaded area indicates the 95% confidence intervals. Diagonal represents chance (area under the curve, AUC = 0.5). **b** Distribution of AUC values across 20 stratified train-test splits (80:20). The red dashed line indicates the mean AUC. **c** Five-fold cross-validation results confirming consistent performance. The red dashed line indicates the mean AUC. **d** Feature importance scores showing the relative contribution to classification. Features are color-coded by category.

The Random Forest classifier demonstrated exceptional performance across multiple validation strategies (Fig. 2). Twenty independent train-test splits yielded a mean area under the curve (AUC) of 0.892 ± 0.075, with five-fold cross-validation demonstrating a mean AUC of 0.882 (Fig. 2a–c). Precision-Recall analysis confirmed robust performance despite class imbalance (68 proctor vs 30 non-proctor), with an average precision of 0.940 (Supplementary Fig. 1).

Because expertise is a surgeon-level attribute, we additionally evaluated generalization to unseen surgeons using leave-one-surgeon-out and surgeon-grouped cross-validation (Supplementary Data 7). A small set of fundamental indicators generalized to unseen surgeons. Models restricted to the three highest-ranked features by importance (left peak density, right speed coefficient of variation, and anastomosis time) reached leave-one-surgeon-out AUC 0.788 (Random Forest) and 0.844 (logistic regression), with surgeon-grouped AUC 0.877 and 0.883, respectively, and left peak density alone, modeled as a single feature, discriminated at AUC 0.869. Because a single feature cannot encode multivariable surgeon-specific patterns, this provides the most leakage-resistant evidence that the expertise-associated signal generalizes across surgeons. By contrast, the full 25-feature Random Forest generalized less well to unseen surgeons (leave-one-surgeon-out AUC 0.571 versus a case-wise estimate of 0.888), indicating that its high case-wise performance partly reflected surgeon-specific patterns. The expertise-associated signal is therefore concentrated in a small set of fundamental indicators rather than in the full multivariable model. Feature importance analysis revealed movement pattern consistency (left peak density, 11.9%) as a highly predictive characteristic, followed by movement variability (right speed coefficient of variation, 11.1%) and anastomosis time (9.0%). The highest-ranked single feature was a left-hand metric (left peak density), consistent with the non-dominant hand's critical role in maintaining tissue tension and needle control during vesicourethral anastomosis (Fig. 2d). These features reflect fundamental movement characteristics that, in theory, can be extracted from any robotic system capable of providing 3D coordinate data.

### Kinematic patterns and clinical outcomes

Partial correlation analysis, controlling for anastomosis time, was conducted on time-independent features to identify associations with clinical outcomes beyond task duration (Table 3, Supplementary Data 6). Left movement sharpness correlated with estimated blood loss (*r* = 0.202, *p* = 0.046), as did left workspace volume (*r* = 0.204, *p* = 0.044). In an exploratory analysis of a small number of patients with anastomotic leaks (*n* = 5), movement-related features exhibited larger effect sizes compared with those in patients without leaks (Table 4). Proctor surgeons achieved shorter console times (227.6 ± 78.4 vs 281.4 ± 76.9 min, *p* = 0.008) with comparable blood loss rates (150.6 ± 187.3 vs 203.0 ± 305.1 mL, *p* = 0.975) (Table 5). At 3 months, the continence recovery rate was 46.2% for proctors compared with 23.3% for non-proctors (*p* = 0.058), with differences diminishing by 6 months (58.5% vs. 53.3%, *p* = 0.805) (Supplementary Fig. 2).

**Table 3:** Partial correlations control for anastomosis time. Analysis identifies efficiency-related features independent of task duration

	Console time				Estimated blood loss	
Feature	Simple *r*	*p*-value	Partial r	*p*-value	*r*	*p*-value
Bilateral Dwell synchrony	−0.303	0.002**	−0.282	0.005**	-	-
Mean hand distance	0.218	0.031*	0.246	0.015*	-	-
Right dwell time	0.114	0.262	−0.138	0.177	-	-
Right speed CV	0.189	0.063	−0.106	0.302	-	-
Left movement sharpness	0.105	0.305	−0.084	0.411	0.202	0.046*
Right peak density	−0.096	0.345	0.077	0.451	-	-
Left peak density	−0.331	<0.001***	0.069	0.503	-	-
Left dwell time	0.244	0.015*	0.068	0.508	-	-
Left speed CV	0.231	0.022*	−0.067	0.514	-	-
Right movement sharpness	0.08	0.433	−0.055	0.595	-	-
Left workspace volume	0.375	<0.001***	−0.053	0.609	0.204	0.044*
Right workspace volume	0.394	<0.001***	0.052	0.614	-	-
Speed Correlation	0.002	0.985	−0.039	0.703	-	-
Motion Synchrony	−0.117	0.25	0.018	0.858	-	-

Bilateral dwell synchrony and mean hand distance remained significant after controlling for anastomosis time, indicating these represent fundamental coordination skills rather than speed-related artifacts.

*r* Pearson correlation coefficient, *Workspace volume* spatial volume occupied by hand motion, .

Partial correlations control for anastomosis time. Only time-normalized and proportional features shown. *p < 0.05, **p < 0.01, ***p < 0.001.

**Table 4:** Large effect sizes for binary outcomes

	Anastomotic leak		Pad-free for 3 m after surgery		Pad-free for 6 m after surgery	
Feature	Effect size	*p*-value	Effect size	*p*-value	Effect size	*p*-value
Left peak density	0.872	0.057	0.016	0.939	0.138	0.615
Left movement sharpness	1.639	<0.001***	0.101	0.408	0.209	0.302
Right movement sharpness	1.825	<0.001***	0.077	0.623	0.166	0.56
Left speed Q25	2.309	<0.001***	0.181	0.529	0.428	0.062
Left speed Mean	2.094	<0.001***	0.136	0.461	0.323	0.157
Right speed mean	1.944	<0.001***	0.091	0.606	0.255	0.271
Left jerk mean	2.067	<0.001***	0.105	0.564	0.302	0.24
Right jerk mean	1.894	<0.001***	0.086	0.623	0.229	0.249
Left path length	1.4	0.011*	0.4	0.098	0.556	0.020*
Right path length	1.312	0.026*	0.387	0.119	0.525	0.027*

*Q25* 25th percentile, *Mean* average across time series, *Jerk* dimensionless jerk.

Features showing Cohen’s *d* > 0.8 for anastomotic leak prediction highlight the discriminative power of movement sharpness and speed metrics in identifying patients at risk for complications.

**Table 5:** Clinical outcomes comparison between proctor and non-proctor surgeons

Outcome	Proctor	Non-Proctor	*p*-value
Console Time (min)	227.6 ± 78.4	281.4 ± 76.9	0.008**
Blood Loss (mL)	150.6 ± 187.3	203.0 ± 305.1	0.975
Leak Rate	5.9% (4/68)	3.3% (1/30)	0.976
Pad-free 3 m	46.2% (30/65)	23.3% (7/30)	0.058
Pad-free 6 m	58.5% (38/65)	53.3% (16/30)	0.805

Leak Rate: proportion of patients experiencing anastomotic leak during hospitalization;

Pad-free 3 m, proportion of patients not using pads at 3 months postoperatively;

Pad-free 6 m, proportion of patients not using pads at 6 months postoperatively.

Proctor surgeons demonstrated superior efficiency (shorter console times), while maintaining safety (similar blood loss), demonstrating that speed need not compromise quality. Leak rate shows no significant difference, while continence recovery at 3 months trended in favor of proctors.

**p < 0.01.

### Heterogeneous learning trajectories define expert adaptation

**Fig. 3:**
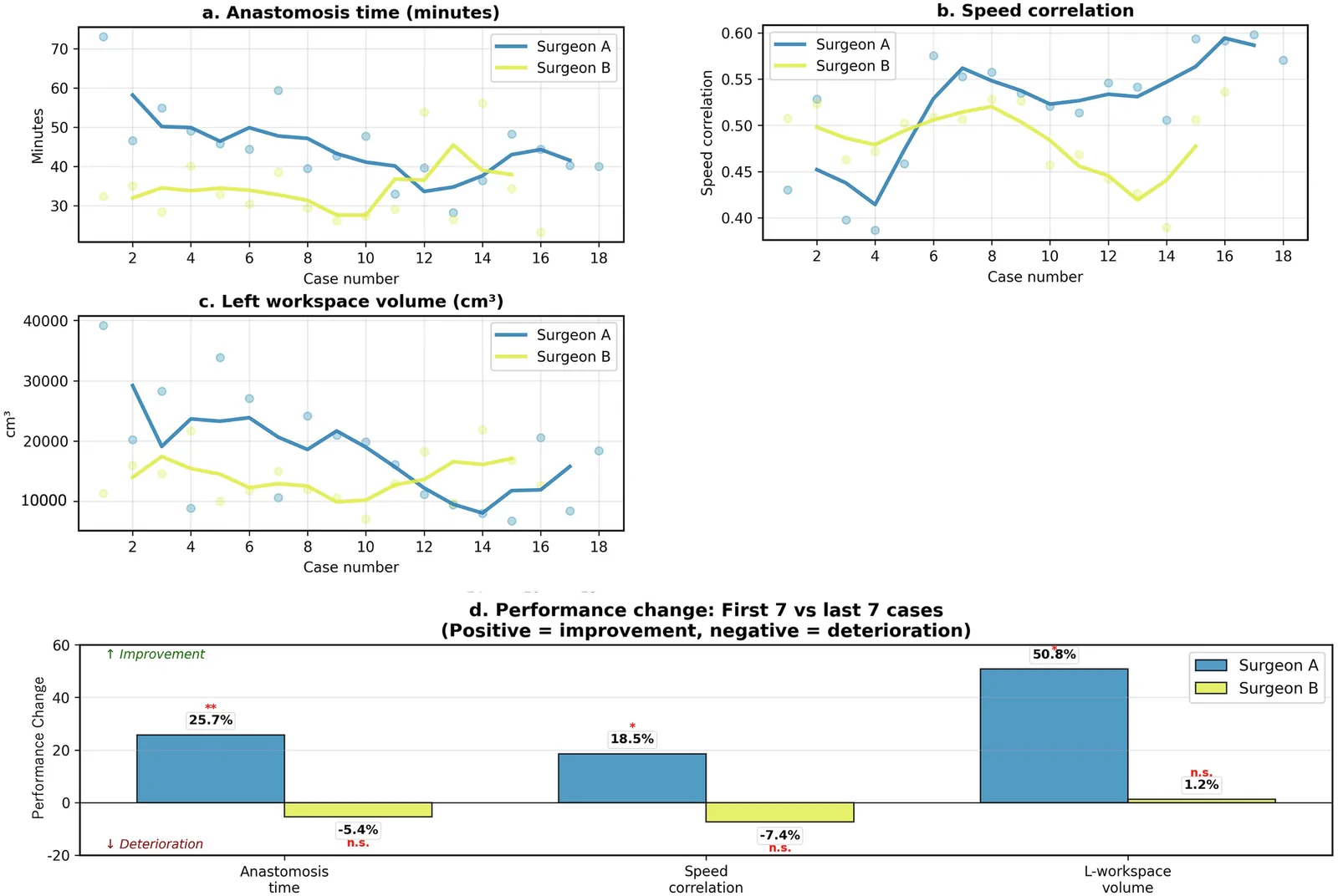
Heterogeneous adaptation patterns among expert surgeons. Two certified proctors demonstrated contrasting learning trajectories on the Hinotori platform. Surgeon A (blue, *n* = 18) exhibits progressive improvement, while Surgeon B (magenta, *n* = 14) shows immediate skill transfer. Lines represent three-case moving averages with individual cases shown as points. **a** Anastomosis time: Surgeon A improves 25.7%, while Surgeon B maintains 30–35 min throughout. **b** Speed correlation: Both improve bimanual coordination, with greater gains observed in Surgeon A. **c** Left workspace volume, an exploratory within-surgeon longitudinal trend based on a bounding-box measure. Surgeon A reduces excessive movement by 50.8%, a change also seen with a 5th to 95th percentile robust estimate (approximately 52.8%), whereas Surgeon B shows little net change. **d** Quantitative comparison of early (first 7 cases) versus late (last 7 cases). Positive values indicate improvement; negative values indicate deterioration.

The two certified proctors exhibited distinct adaptation patterns on the Hinotori platform (Fig. 3). Surgeon A (*n* = 18) showed progressive changes, with a 25.7% reduction in anastomosis time, an 18.5% increase in speed correlation, and a 50.8% decrease in left workspace volume. Surgeon B (*n* = 14) maintained consistent performance metrics from the first case. The greatest differences between the two surgeons were observed in bimanual coordination metrics. Surgeon A improved bilateral dwell time by 8.7%, while Surgeon B showed a 4.8% increase. For speed correlation, Surgeon A demonstrated an 18.5% improvement, whereas Surgeon B exhibited a 7.4% decrease.

### Sensitivity analyses

Sensitivity analysis assessing model robustness to individual surgeon effects confirmed stable classification performance across different surgeon inclusion/exclusion scenarios, with AUC values ranging from 0.86 to 0.91 (Supplementary Fig. 3). Performance was maintained even when excluding the highest-volume non-proctor surgeon who contributed 16 of the 30 non-proctor cases (AUC = 0.86), indicating that the case-wise result is not attributable to any single dominant surgeon. This inclusion/exclusion analysis (Supplementary Fig. 3) is distinct from the surgeon-held-out validation reported above and does not by itself assess generalization to unseen surgeons. Excluding the jerk-based features changed case-wise performance only minimally (delta AUC approximately 0.01), consistent with their strong correlation with task duration and path length, which left little information independent of the retained features. Power analysis demonstrated 95.1% power to detect effect sizes with *d* > 0.8. The mean observed effect size among significant features was 0.94 (Supplementary Fig. 4, Supplementary Data 5).

## Discussion

In this study, it was established that fundamental movement patterns derived from three-dimensional coordinates provide sufficient information for accurate surgical skill assessment, achieving performance comparable to that of comprehensive platform-specific approaches while offering potential applicability across platforms with position-tracking capability. Our findings highlight three key insights: (1) movement pattern consistency (peak density) and smoothness serve as robust kinematic indicators of surgical expertise; (2) machine learning based on just 25 kinematic features can accurately classify expertise levels; and (3) expert surgeons display heterogeneous adaptation patterns when transitioning between platforms.

The concentration of discriminative power in movement patterns, particularly peak density (d = 1.618), is consistent with established motor-learning principles^1,20–22^. Peak density was defined operationally as the frequency of local peaks in the hand-speed profile. We do not interpret higher peak density as skillful rhythmicity in isolation, because repetitive velocity peaks could also arise from mechanical or strategically inefficient motion. During vesicourethral anastomosis these peaks plausibly correspond to recurring submovements such as needle driving, suture handling, and hand repositioning, although coordinate-only data do not segment these gestures directly. Peak density partially overlapped with efficiency (correlation with task time, Spearman rho = −0.53) but was not reducible to task time alone. After adjustment for task time, it retained discrimination across unseen surgeons (residualized leave-one-surgeon-out AUC 0.771, partial correlation with expertise *r* = 0.45, *p* < 0.001), and combining it with task time improved unseen-surgeon discrimination from 0.869 to 0.885. We therefore interpret repeated movement structure together with efficiency rather than rhythmicity alone. We speculate that the observed pattern of reduced pausing (lower dwell time) combined with consistent speed (lower CV) in expert surgeons may reflect what has been described as ‘flow state’ in expertise research, though this interpretation remains conjecture requiring dedicated psychological assessment in future studies. Our finding that left peak density exhibited the strongest effect size likely reflects the non-dominant hand’s critical role in maintaining tissue tension and needle control during anastomosis.

Our approach, which relies solely on fundamental kinematic features, offers advantages over existing comprehensive assessment methods. Hung et al.^2^ achieved comparable discrimination between expert groups using over 40 automated performance metrics from the da Vinci system, including camera movements, clutch usage, and energy application patterns. Conversely, while the full 25-feature model matched comprehensive approaches in case-wise discrimination (AUC 0.892), it did not generalize to unseen surgeons; the generalizable signal was concentrated in a few fundamental indicators. This parsimony is essential as robotic systems proliferate globally and movement patterns, such as peak density and smoothness, reflect

fundamental neuromuscular control patterns that may transcend specific interfaces, whereas system-specific events can vary across platforms and software versions^12,13,22,23^. However, the availability of raw coordinate data varies across platforms, and further validation is needed to confirm the cross-platform applicability of our approach. Our coordinate-derived indicators should be interpreted as complementary to, rather than replacements for, expert video-based assessment tools such as GEARS. GEARS evaluates multidimensional robotic skill through expert review, including depth perception, bimanual dexterity, efficiency, force sensitivity, autonomy, and robotic control. In prior dVLogger work during robot-assisted radical prostatectomy, expertise was defined using case-volume thresholds, and blinded GEARS scoring was used for concurrent validation. During the vesicourethral anastomosis, that work found domain-specific concordance between automated kinematic metrics and GEARS, with time- and velocity-related metrics showing moderate associations with the GEARS efficiency domain^5,24^. These findings indicate that automated kinematic indicators and GEARS capture overlapping but non-identical constructs. Accordingly, we do not claim GEARS-level convergent validity for the present retrospective coordinate-only dataset. Future prospective studies should include blinded multi-rater GEARS assessment with inter-rater reliability analysis to define how coordinate-derived indicators map onto expert-rated technical domains.

The strong correlation between anastomosis time and total console time (*r* = 0.704, *p* < 0.001) warrants special consideration. Vesicourethral anastomosis represents only one phase of robot-assisted radical prostatectomy; however, we interpret it as an indicator of performance within this specific suturing-dominant phase rather than a proxy for overall procedural proficiency. This relationship likely reflects that surgeons who struggle with the technically demanding anastomosis phase may also encounter difficulties in other complex stages, such as nerve sparing or apical dissection^25–27^. The anastomosis phase offers distinct advantages for standardized assessment, as it requires consistent bimanual coordination, follows a predictable sequence, and directly influences functional outcomes. These characteristics make it a standardized, suturing-dominant phase suitable for objective, phase-specific skill evaluation^28–30^, enabling targeted assessment without the need to analyze entire procedures.

The association between movement quality metrics and clinical outcomes enables performance monitoring in practical settings. Institutions could adopt kinematic thresholds as preliminary benchmarks for future validation: maintaining a left peak density above 0.082, dimensionless jerk below 5.5 × 10^5^, and speed coefficient of variation below 1.10 aligns with proctor-level performance (Supplementary Data 4). These thresholds should be considered hypothesis-generating and require prospective validation before clinical application. The correlations between kinematic features and blood loss, while statistically significant (path length: *r* = 0.33, *p* < 0.001; jerk: *r* = 0.26, *p* < 0.01), are modest in magnitude, explaining approximately 7–11% of variance. This suggests that movement patterns during urethrovesical anastomosis have a detectable but limited association with overall blood loss. This modest effect size is clinically plausible, as the anastomosis phase represents only a portion of the total procedure, and blood loss is influenced by multiple factors including prostate vascularity and patient anatomy^31,32^. Shorter console times without increased complications indicate that efficiency does not compromise safety, a critical finding for training programs^33,34^. While our exploratory analysis of anastomotic leak cases (*n* = 5) revealed large effect sizes for movement features, the small sample size limits definitive conclusions. Our identification of distinct learning phenotypes among expert surgeons transitioning to the Hinotori platform represents a novel contribution of this study. The contrast between the progressive adapter pattern (Surgeon A: 25.7% improvement in anastomosis time, 50.8% reduction in workspace volume) and the immediate transfer pattern (Surgeon B: consistent performance from case one) challenges the assumption of uniform skill transfer among experts. The differential improvement in bimanual coordination ( + 18.5%) compared with the unilateral skills suggests platform-specific optimization requirements, even for experienced surgeons. This heterogeneity has important implications for platform adoption strategies. Institutions should consider implementing baseline kinematic assessments to identify surgeons who may require additional support during transitions.

Our approach, based on fundamental kinematic features, has the potential to democratize access to objective performance evaluation. As robotic systems proliferate globally, from China’s Micro Hand S^6–8^ to Europe’s Versius^10^ and Hugo^11,35^ platforms, the need for platform-agnostic assessment methods has become critical. By focusing on fundamental movements rather than on proprietary metrics, institutions with access to basic position-tracking data can potentially implement meaningful skill evaluations. This accessibility is especially important for emerging markets, where access to advanced analytics for specific platforms may be limited, yet the need for objective surgical assessment remains critical. An additional methodological analysis in this study was the explicit evaluation of generalization to unseen surgeons. Many prior automated performance metric studies used case-level validation designs appropriate to their original aims. Because kinematic patterns can encode surgeon-specific tendencies, we additionally applied surgeon-held-out validation, which provides a more stringent test of whether the expertise-associated signal transfers beyond the surgeons used for model development. To our knowledge, few coordinate-derived robotic skill studies have explicitly reported surgeon-held-out validation. This analysis showed that the high-dimensional classifier did not generalize on its own, whereas a small set of fundamental, interpretable indicators retained discrimination across unseen surgeons.

This study has certain limitations that warrant consideration. First, the analysis was confined to vesicourethral anastomosis, a single suturing-dominant phase performed at one institution on one robotic platform. It therefore does not capture tissue dissection, energy-device handling, or intraoperative judgment, and characterizes phase-specific performance rather than overall surgical proficiency. Validation across additional surgical phases, institutions, and robotic platforms is needed before claims of platform independence can be substantiated. Second, the correlation between dimensionless jerk and duration, although addressed through partial correlation analysis, warrants investigation using alternative smoothness metrics. The 1 Hz sampling frequency is well below that required for reliable third-order derivatives (30–60 Hz). Consequently, our jerk-based features are a coarse surrogate for movement smoothness and should be regarded as exploratory, and our principal conclusions accordingly rest on peak density, task time, and speed variability rather than on jerk-based measures. Third, the small number of anastomotic leak cases (*n* = 5) limits the reliability of the complication findings, although the observed effect sizes motivate investigation in larger cohorts. Fourth, expertise was defined by proctor certification rather than by expert video-based rating, and prospective convergent validation against established global rating scales such as GEARS remains a priority for future work. Finally, the parsimonious models were derived from 16 right-handed surgeons, and these results, together with long-term oncological outcomes, require prospective external validation in larger and mixed-handedness cohorts before any credentialing or training use. Future studies should focus on developing real-time intraoperative feedback, validating these findings across multiple platforms and surgical specialties, as well as conducting randomized trials of kinematic-based training interventions. Additionally, establishing international benchmarks for kinematic performance could help standardize surgical training globally. Validation on publicly available surgical skill datasets would enable direct comparison with other assessment methods.

In conclusion, kinematic analysis based solely on fundamental three-dimensional position data distinguished proctor-level performance, and a small set of interpretable indicators generalized to unseen surgeons. Because these indicators derive from position coordinates alone, they offer a potential route to platform-independent skill assessment that does not rely on proprietary metrics. Proctor-level performance was associated with greater operative efficiency, whereas associations with specific perioperative outcomes were exploratory and require confirmation. Prospective, multi-surgeon validation is needed before clinical deployment.

## Methods

### Study design and participants

**Fig. 4:**
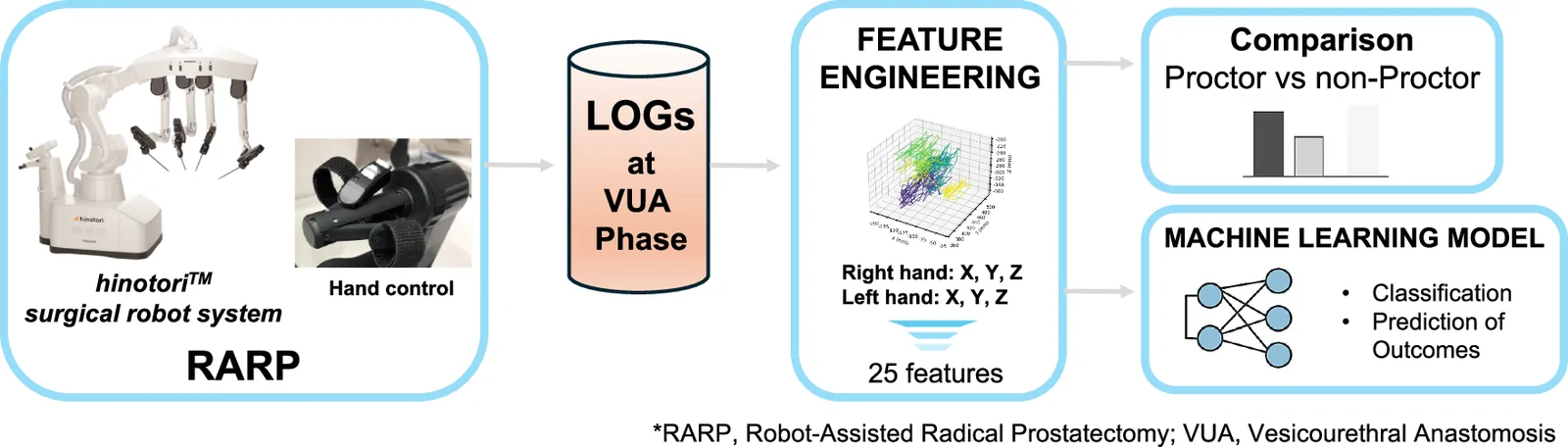
Study design and data processing workflow. The Hinotori Surgical Robot System captures three-dimensional coordinate data from bilateral hand controllers during robot-assisted radical prostatectomy. Raw positional data (x, y, z) is extracted from operation logs and processed through a feature engineering pipeline to generate 25 kinematic features across five domains. These features are used to compare proctor and non-proctor surgeons and serve as inputs for machine learning classification. RARP Robot-Assisted Radical Prostatectomy, VUA Vesicourethral Anastomosis.

In this retrospective observational study, we analyzed consecutive robot-assisted radical prostatectomies performed using the Hinotori Surgical Robot System (Medicaroid Corporation, Kobe, Japan) between December 2020 and November 2023 at Kobe University Hospital (Fig. 4). The study protocol was approved by the Kobe University Hospital Institutional Review Board (approval number: B230172) in accordance with the Declaration of Helsinki. The requirement for written informed consent was waived by the Kobe University Hospital Institutional Review Board because this study was a retrospective analysis of routinely collected clinical and operative data, and all data were anonymized prior to analysis. Of 132 consecutive procedures, 98 (74.2%) had complete kinematic logs during vesicourethral anastomosis. The remaining 34 cases were excluded owing to technical logging failures, primarily network connectivity issues, during early adoption.

### Surgeon stratification

Surgeons were classified as ‘Proctor’ (expert) or ‘Non-Proctor’ based on formal Proctor certification for robot-assisted urologic surgery in Japan. Proctor certification requires: (1) Board certification as a Japanese Urological Association certified specialist, (2) Membership in the Japanese Society of Endourology and Robotics, (3) ≥ 40 cases as primary surgeon (radical prostatectomy and/or radical cystectomy combined), (4) At least one publication or conference presentation on robot-assisted surgery, and (5) Completion of the official Proctor education seminar. This certification-based definition represents a rigorous, multi-dimensional credentialing standard that incorporates case volume, academic engagement, and formal training assessment. Certified proctors performed 68 procedures (69.4%) and non-proctors 30 procedures (30.6%). All surgeons were right-handed. Detailed profiles are provided in Supplementary Data 2.

### Kinematic data acquisition and processing

The Hinotori system records three-dimensional position data at 1 Hz using the Medicaroid Intelligent Network System. We extracted the bilateral hand controller coordinates (x, y, and z in millimeters) during vesicourethral anastomosis, defined as the interval from the first needle grasp to final knot completion. Missing data ( < 0.1%) were addressed using bidirectional filling. The raw operation log files contain time-stamped three-dimensional Cartesian coordinates (X, Y, Z in millimeters) for each hand controller. Preprocessing included forward-fill followed by backward-fill for missing values, a minimum trajectory length requirement of 100 samples (100 s), and truncation of left and right trajectories to equal length.

### Feature engineering framework

We developed a comprehensive feature extraction pipeline that generated 25 kinematic parameters across five categories, all derived solely from 3D position coordinates, making them theoretically extractable from any robotic system with position tracking capability (Table 6): *Movement Smoothness*: Dimensionless jerk, and jerk mean.

**Table 6:** Comprehensive kinematic features extracted from bilateral hand position data

No.	Category	Feature	Description	Unit
1	Movement Smoothness	Left dimensionless jerk	Scale-invariant measure of left hand movement smoothness	dimensionless
2	Movement Smoothness	Right dimensionless jerk	Scale-invariant measure of right hand movement smoothness	dimensionless
3	Movement Smoothness	Left jerk mean	Mean jerk magnitude of left hand	mm/s³
4	Movement Smoothness	Right jerk mean	Mean jerk magnitude of right hand	mm/s³
5	Speed Characteristics	Left speed mean	Average velocity of left hand	mm/s
6	Speed Characteristics	Right speed mean	Average velocity of right hand	mm/s
7	Speed Characteristics	Left speed CV	Coefficient of variation of left hand speed	dimensionless
8	Speed Characteristics	Right speed CV	Coefficient of variation of right hand speed	dimensionless
9	Speed Characteristics	Left speed 25th percentile	25th percentile of left hand speed distribution	mm/s
10	Speed Characteristics	Right speed 25th percentile	25th percentile of right hand speed distribution	mm/s
11	Movement Pattern	Left dwell time	Proportion of time left hand velocity < 2 mm/s	%
12	Movement Pattern	Right dwell time	Proportion of time right hand velocity < 2 mm/s	%
13	Movement Pattern	Left peak density	Number of velocity peaks per second for left hand	peaks/s
14	Movement Pattern	Right peak density	Number of velocity peaks per second for right hand	peaks/s
15	Movement Pattern	Left movement sharpness	Average prominence of velocity peaks for left hand	mm/s
16	Movement Pattern	Right movement sharpness	Average prominence of velocity peaks for right hand	mm/s
17	Workspace Utilization	Left workspace volume	Volume of 3D bounding box for left hand positions	cm³
18	Workspace Utilization	Right workspace volume	Volume of 3D bounding box for right hand positions	cm³
19	Path Characteristic	Left path length	Total distance traveled by left hand	mm
20	Path Characteristic	Right path length	Total distance traveled by right hand	mm
21	Bimanual Coordination	Speed correlation	Pearson correlation between left and right hand speeds	Pearson’s r
22	Bimanual Coordination	Mean hand distance	Average Euclidean distance between hands	mm
23	Bimanual Coordination	Motion synchrony	Sliding window correlation of hand movements	correlation index
24	Bimanual Coordination	Bilateral Dwell synchrony	Proportion of time both hands simultaneously static	%
25	Temporal	Anastomosis time	Total duration of urethrovesical anastomosis phase	minutes

Features were organized into five functional categories reflecting different aspects of surgical performance. Movement smoothness quantifies the control quality using jerk-based metrics. Speed characteristics capture velocity patterns and consistency. Movement patterns were used to identify periods of stillness. Workspace utilization was used to measure three-dimensional space usage efficiency. Path efficiency relates actual to optimal trajectories. Bimanual coordination assesses inter-hand synchrony. Each feature’s unit and clinical interpretation were provided to facilitate implementation across robotic platforms.

*Speed Characteristics*: Mean velocity, coefficient of variation, and percentile distributions (25th).

*Movement Patterns*: Dwell-time percentage (velocity <2 mm/s) and peak density in the velocity profile.

*Workspace Utilization*: Three-dimensional bounding box volume and range of motion.

*Path Efficiency*: Ratio of Euclidean distance to actual path length.

*Bimanual Coordinatio*n: Interhand speed correlation, mean distance, motion synchrony, and bilateral dwell synchrony.

Complete mathematical definitions are provided in Supplementary Data 4. Velocity was computed as the first-order difference of consecutive position coordinates divided by the sampling interval (Δt = 1 s). Speed was defined as the Euclidean norm of the velocity vector. Dimensionless jerk was calculated as DJ = √((1/2)·mean(jerk^2^) × T^5^/L^2^), where jerk is the third-order difference of position, mean(jerk^2^) is the mean squared jerk magnitude across samples, T is the duration in seconds, and L is the total path length in millimeters. Mean jerk was the average of jerk magnitudes. Peak density was defined as the number of peaks in the speed profile per unit time (peaks/s), identified using scipy.signal.find_peaks with height, distance, and prominence thresholds derived from each trajectory’s speed distribution. Dwell time was the proportion of samples with speed below 2.0 mm/s. Workspace volume was calculated as the three-dimensional bounding box volume (range_X × range_Y × range_Z). Path efficiency was the ratio of the Euclidean distance between the start and end positions to the total path length. Bimanual coordination metrics included: interhand speed correlation (Pearson correlation between left and right hand speeds over the entire time series without windowing), motion synchrony (the mean of Pearson correlations between left- and right-hand speed profiles computed over consecutive, non-overlapping windows of 50 samples, corresponding to 50-s windows at the sampling rate), mean bimanual distance (average Euclidean distance between hands), and bilateral dwell synchrony (proportion of time both hands had speed below the 10th percentile of their respective speed distributions).

### Statistical analysis and reproducibility

All statistical analyses were conducted using Python 3.8, employing scipy.stats and scikit-learn libraries. Regarding between-group comparisons, we used two-tailed Mann–Whitney *U* tests with Benjamini–Hochberg false discovery rate correction (α = 0.05). Effect sizes were calculated using Cohen’s d, based on pooled standard deviations. Data are presented as mean ± standard deviation unless otherwise specified. For correlation analyses, Spearman’s rank correlation was used, as non-normal distributions were confirmed using Shapiro–Wilk tests. All *p*-values are reported exactly. Stratified sampling was used to maintain class balance in all machine learning models. Error bars in the figures represent the standard error of the mean. Partial correlations were controlled for anastomosis time to identify efficiency-independent features.

To assess model robustness to individual surgeon effects, we performed sensitivity analysis evaluating classification performance under different surgeon inclusion/exclusion conditions. For each scenario (all data, without highest-volume non-proctor surgeon contributing 16/30 non-proctor cases, etc.), we conducted 10 iterations of stratified 80/20 train-test splits and computed mean AUC. To assess generalization to unseen surgeons, we additionally performed leave-one-surgeon-out and surgeon-grouped (five-fold) cross-validation, holding out all cases from a given surgeon as the test set. Because most surgeons contributed cases of a single expertise class, we report pooled out-of-fold AUC, with feature standardization fit within each training fold. We further evaluated parsimonious models restricted to the three highest-importance features (Fig. 2d) using both Random Forest and logistic regression. To evaluate the impact of the 1 Hz sampling limitation on jerk-based features, we compared classification performance with and without jerk-based features using the same classification pipeline (20 stratified train-test splits, Random Forest with class_weight = ‘balanced’).

### Machine learning implementation

Random Forest classification was implemented using scikit-learn, with hyperparameter optimization performed via five-fold grid search. Parameters included: n_estimators=100, max_depth=None, min_samples_split=5, min_samples_leaf=2, class_weight = ‘balanced’. Random Forest was selected for the main analysis because it: (1) provides feature importance rankings for interpretability, (2) shows robust performance across cross-validation and test sets, and (3) handles potential non-linear relationships without explicit specification. Performance was evaluated using the area under the receiver operating characteristic curve across 20 iterations with different random seeds. Feature importance was determined based on the mean decrease in Gini impurity. Alternative classifiers (SVM, Neural Network, Logistic Regression, Gradient Boosting) were systematically compared (Supplementary Data 3). Feature preprocessing included z-score normalization using StandardScaler. Cross-validation used stratified 5-fold splits (shuffle=True, random_state=42).

### Clinical outcome extraction

Perioperative outcomes (including console time, blood loss, anastomotic leak, and continence recovery [pad-free at 3/6 months]) were extracted from medical records. Association analyses were conducted using appropriate statistical tests, with clinical thresholds defined in Supplementary Data 4.

### Software environment

All analyses were performed using Python 3.8 with the following packages: numpy ( ≥ 1.24) for numerical computation, pandas ( ≥ 2.0) for data manipulation, scipy ( ≥ 1.10) for signal processing and statistical tests, scikit-learn ( ≥ 1.2) for machine learning, and matplotlib ( ≥ 3.7) for visualization.

## Supplementary information

Supplementary Information

Supplementary_Data_1.

Supplementary_Data_2.

Supplementary_Data_3.

Supplementary_Data_4.

Supplementary_Data_5.

Supplementary_Data_6.

Supplementary_Data_7.

## Data Availability

The datasets generated and analyzed during the current study are not publicly available due to privacy and ethical restrictions, as movement patterns could potentially identify individual surgeons, but are available from the corresponding author on reasonable request. Researchers interested in accessing the data should contact the corresponding author (H.U., hideueki@med.kobe-u.ac.jp) to discuss data-sharing agreements that ensure appropriate privacy protection and comply with the ethical approval granted by the Kobe University Hospital Institutional Review Board (approval number: B230172).

## Code availability

The custom Python code for kinematic feature extraction, expertise classification, and surgeon-aware validation, which reproduces the central findings of this study, will be made available in a public repository with a permanent DOI upon publication. The released code does not include patient-identifiable raw surgical logs. The code was developed using Python 3.8 with the following key dependencies: numpy (≥1.24), pandas (≥2.0), scipy (≥1.10), scikit-learn (≥1.2), and matplotlib (≥3.7). The code includes: (1) feature extraction functions for all 25 kinematic metrics with the mathematical definitions described in the Methods, (2) machine learning pipeline with hyperparameter configurations (Random Forest: n_estimators=100, max_depth=None, class_weight = ‘balanced’, random_state=42), and (3) visualization scripts for generating all figures. No custom software or specialized hardware is required beyond standard Python scientific computing packages.
