# Supplementary material for: Fundamental kinematic indicators predict surgical expertise and clinical outcomes in robot-assisted surgery
Source: NPJ Digit Med. 2026 Sep 29;9:736. doi: 10.1038/s41746-026-03137-9 (PMC13623960; doi:10.1038/s41746-026-03137-9)
Supplement: Supplementary file 1 [file 41746_2026_3137_MOESM1_ESM.pdf]

Supplementary Information

Fundamental kinematic indicators predict surgical expertise and clinical outcomes in robot-assisted surgery

Hideto Ueki, Takuto Hara, Tomoko Yamaguchi, Naoto Wakita, Yasuyoshi Okamura, Kotaro Suzuki, Yukari Bando, Kiyoyuki Chinzei, Munenori Uemura, Tomoaki Terakawa, Akihisa Yao, Jun Teishima, Koji Chiba, Hideaki Miyake

Table of Contents

• Supplementary Figure 1: ROC and Precision-Recall curves
• Supplementary Figure 2: Continence recovery rates
• Supplementary Figure 3: Surgeon exclusion sensitivity analysis
• Supplementary Figure 4: Power analysis

• Supplementary Data 1–7 are provided as separate Excel files.

• STROBE Checklist

Supplementary Figure 1

Receiver operating characteristic (ROC) and Precision-Recall (PR) curves for the Random Forest classifier. (a) ROC curve for a representative stratified train-test split (80:20). The solid line shows the classifier performance (AUC=0.875); the shaded area represents the area under the curve. The dashed diagonal line represents chance performance (AUC=0.5). The red dot indicates the optimal operating point (threshold=0.8; true positive rate=0.70, false positive rate=0.07). (b) Precision-Recall curve confirming robust classification performance despite class imbalance (68 proctor vs 30 non-proctor). The average precision (AP) was 0.940, indicating high precision across a wide range of recall values. The dashed horizontal line represents the precision of a random classifier (0.70, reflecting the class prior). The red dot indicates the optimal F1 operating point (threshold=0.5; precision=0.85, recall=0.93). Across 20 independent splits, the mean AUC was 0.892 ± 0.075.

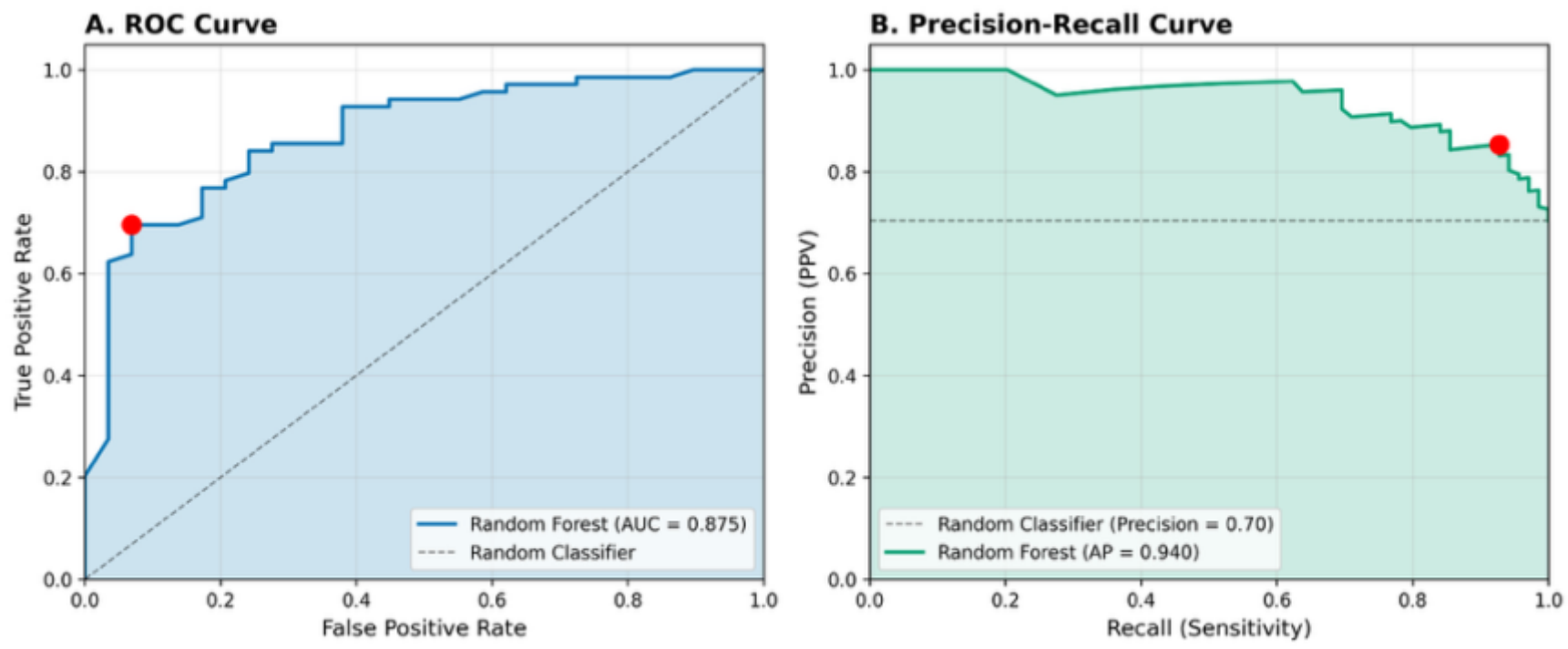

Supplementary Figure 2

Continence recovery rates after robot-assisted radical prostatectomy. Comparison of pad-free continence recovery between proctor (blue circles, n=65) and non-proctor (magenta squares, n=30) surgeons at 3 and 6 months postoperatively. Three proctor cases were excluded owing to missing continence follow-up data. At 3 months, proctor surgeons achieved a higher continence recovery rate (46%) compared with non-proctor surgeons (23%), with a trend toward significance (p=0.06, chi-square test). By 6 months, the difference diminished (58% vs 53%, p=0.81), suggesting that initial expertise-related advantages in functional outcomes converge over the longer recovery period. Error bars represent 95% confidence intervals.

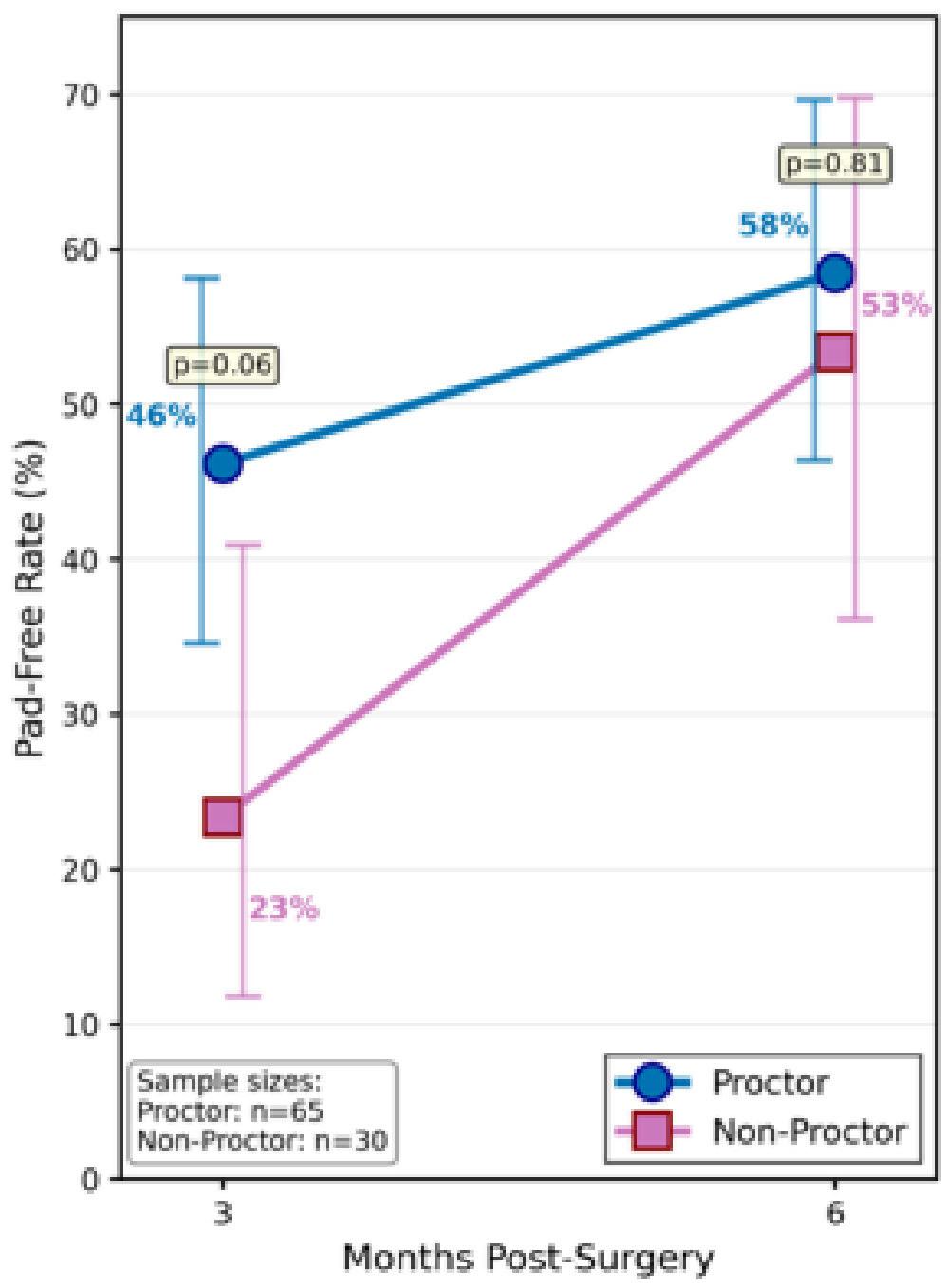

Supplementary Figure 3

Sensitivity analysis of classification performance across surgeon inclusion/exclusion scenarios. To assess model robustness to individual surgeon effects, Random Forest classification was evaluated under four conditions: all data (N=98), without the highest-volume non-proctor surgeon who contributed 16 of 30 non-proctor cases (N=82), highest-volume non-proctor versus all proctors (N=84), and remaining non-proctors versus all proctors (N=82). For each scenario, 10 iterations of stratified 80/20 train-test splits were performed. Bar heights represent mean AUC values; error bars indicate standard deviation. Classification performance remained stable (AUC 0.86–0.91) across all scenarios, confirming that findings are not driven by any single surgeon's contribution to the dataset.

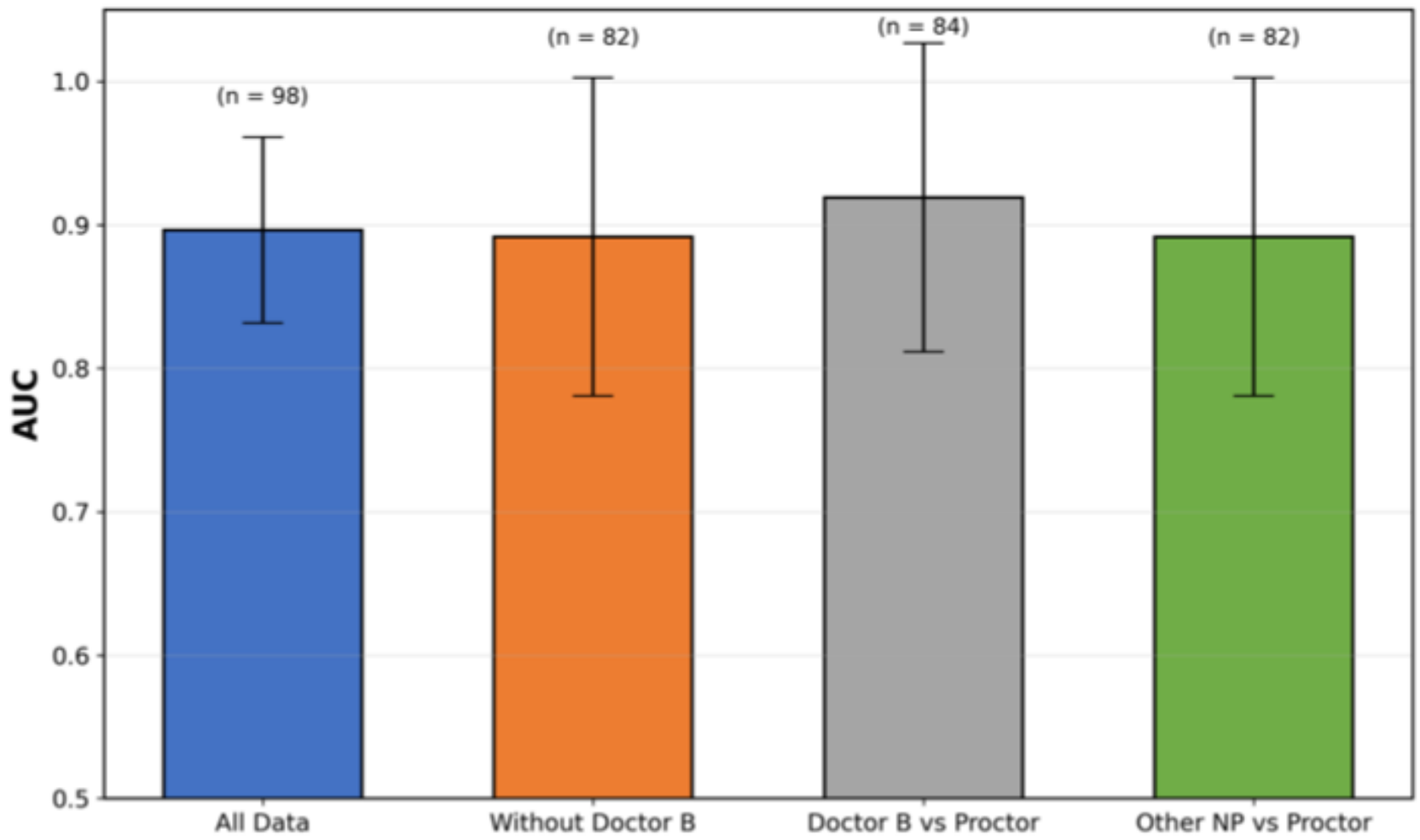

Supplementary Figure 4

Power analysis for detecting differences in robotic surgical skills. Post hoc power analysis comparing the current study design (blue solid line; N=98, 68 proctor vs 30 non-proctor) with hypothetical balanced (green dashed line; N=100, 50:50) and larger (red dotted line; N=200, 100:100) study designs. The horizontal dashed line indicates 80% power (conventional threshold); the vertical dashed line indicates Cohen's d=0.8 (large effect size). The orange vertical line marks the mean observed effect size (|d|=0.94) among the 15 statistically significant features. At d=0.8, the current study achieves 95.1% power, confirming adequate statistical power for the primary between-group comparisons.

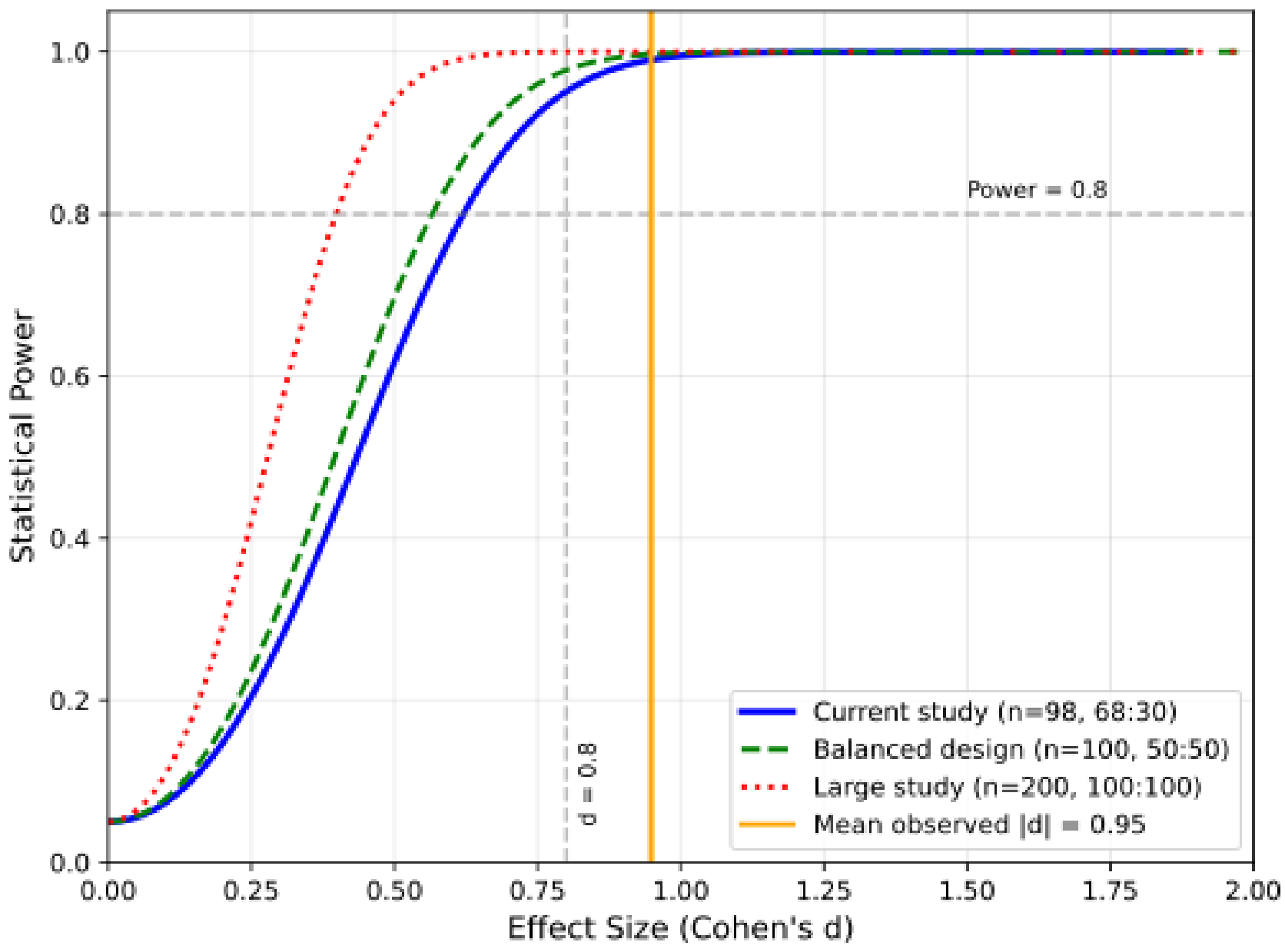

Supplementary Data 1

Complete kinematic feature comparison between proctor and non-proctor surgeons. All 25 kinematic features extracted from bilateral hand position data during vesicourethral anastomosis, with detailed descriptive statistics for proctor (n=68) and non-proctor (n=30) groups. Values are presented as mean ± standard deviation. Statistical comparisons were performed using the Mann–Whitney U test with Benjamini–Hochberg false discovery rate (FDR) correction at $\alpha = 0.05$. Effect sizes are reported as Cohen's d based on pooled standard deviations, interpreted as small (0.2), medium (0.5), large (0.8), and very large (>1.2). Fifteen of 25 features (60%) achieved statistical significance after FDR correction.

[See Supplementary Data 1]

Supplementary Data 2

Surgeon profiles and case distribution. Detailed characteristics of the 16 surgeons who performed the 98 robot-assisted radical prostatectomy procedures, including certification status (proctor/non-proctor), number of cases performed during the study period, prior robotic surgery experience, and specialty training background. All surgeons in this cohort were right-handed.

[See Supplementary Data 2]

Supplementary Data 3

Comparison of machine learning classifiers for expertise classification. Performance of five classification models evaluated using stratified 5-fold cross-validation and 20 independent train-test splits (80:20). Models compared: Random Forest, Support Vector Machine (SVM), Neural Network, Logistic Regression, and Gradient Boosting. All models used class_weight='balanced' to account for the imbalanced class distribution (68 proctor vs 30 non-proctor).

[See Supplementary Data 3]

Supplementary Data 4

Kinematic feature definitions, units, and proposed clinical thresholds. Mathematical definitions and clinical interpretation for all 25 kinematic features organized by functional category (spatial efficiency, movement smoothness, speed characteristics, coordination, and temporal patterns). Velocity was computed as the first-order difference of consecutive position coordinates (numpy.diff). Motion synchrony was defined as the mean of Pearson correlations between the left- and right-hand speed profiles computed over consecutive, non-overlapping windows of 50 samples. Dimensionless jerk was computed from the mean squared jerk magnitude, normalized by duration and path length. Proposed clinical thresholds for proctor-level performance are included as preliminary benchmarks for future validation.

[See Supplementary Data 4]

Supplementary Data 5

Supplementary Data 5. Effect sizes and statistical significance for all kinematic features. Detailed statistical results ranked by absolute Cohen's d effect size. The mean observed effect size among the 15 statistically significant features was 0.94. Power analysis confirmed 95.1% power to detect effects of $d > 0.8$.

[See Supplementary Data 5]

Supplementary Data 6

Correlations between kinematic features and clinical outcomes. Spearman rank correlations (unadjusted) and partial correlations (adjusted for anastomosis time) between time-independent kinematic features and clinical outcomes (estimated blood loss, console time, length of hospital stay). N=98 for all analyses.

[See Supplementary Data 6]

Supplementary Data 7

Surgeon-aware cross-validation of expertise classification. Panel A reports classification performance under case-wise stratified five-fold cross-validation compared with surgeon-grouped five-fold and leave-one-surgeon-out cross-validation, which keep all cases from a given surgeon within a single fold so that every test case comes from a surgeon not seen during training. Values are pooled out-of-fold areas under the receiver operating characteristic curve. The full 25-feature Random Forest did not generalize to unseen surgeons (leave-one-surgeon-out AUC 0.571), whereas models restricted to the top-importance indicators and left peak density alone retained discrimination (leave-one-surgeon-out AUC 0.788 to 0.869). Panel B re-evaluates the same alternative classifiers reported in Supplementary Data 3 under leave-one-surgeon-out cross-validation, in which no multifeature model exceeded the single left peak density indicator and performance did not consistently improve with additional features or nested hyperparameter tuning. Because 15 of the 16 surgeons contributed cases of a single expertise class, the pooled out-of-fold AUC is reported rather than the mean of per-fold values.

[See Supplementary Data 7]

STROBE Statement—Checklist of items for observational studies

Item	No.	Recommendation / Location	Reported
Title and abstract	1	(a) Study design indicated in title/abstract — Title, Abstract<br>(b) Informative summary provided — Abstract	Yes
Background/rationale	2	Scientific background and rationale explained — Introduction	Yes
Objectives	3	Specific objectives stated — Introduction, paragraph 3	Yes
Study design	4	Key elements presented early — Methods, Study Design and Participants	Yes
Setting	5	Setting, locations, relevant dates described — Methods, Study Design	Yes
Participants	6	Eligibility criteria, selection methods given — Methods, Study Design; Surgeon Stratification	Yes
Variables	7	Outcomes, exposures, confounders defined — Methods, Feature Engineering; Clinical Outcome Extraction	Yes
Data sources	8	Sources and methods of assessment detailed — Methods, Kinematic Data Acquisition; Feature Engineering	Yes
Bias	9	Efforts to address bias described — Discussion, limitations; Sensitivity Analyses	Yes
Study size	10	Study size explained — Methods (132 consecutive, 98 included); Supplementary Figure 4	Yes
Quantitative variables	11	Handling of variables explained — Methods, Feature Engineering; Statistical Analysis	Yes
Statistical methods	12	(a) All methods described — Methods, Statistical Analysis; ML Implementation<br>(b) Subgroups examined — Sensitivity Analyses<br>(c) Missing data addressed — Methods (<0.1%)<br>(d) Sampling strategy — Methods (stratified)<br>(e) Sensitivity analyses — Results; Supplementary Figures 3-4	Yes

Participants	13	(a) Numbers at each stage reported — Results; Figure 4<br>(b) Reasons for non-participation — Methods (technical failures)	Yes
Descriptive data	14	(a) Participant characteristics given — Table 1<br>(b) Missing data indicated — Methods (<0.1%)	Yes
Outcome data	15	Outcome events/summary measures reported — Tables 3–5	Yes
Main results	16	(a) Unadjusted and adjusted estimates — Tables 2–5; Supplementary Data<br>(b) Category boundaries reported — Supplementary Data 4<br>(c) Clinical interpretation — Discussion	Yes
Other analyses	17	Other analyses reported — Sensitivity analyses; Learning curves	Yes
Key results	18	Key results summarised — Discussion, paragraph 1	Yes
Limitations	19	Limitations discussed — Discussion, limitations paragraph	Yes
Interpretation	20	Cautious interpretation given — Discussion, final paragraphs	Yes
Generalisability	21	Generalisability discussed — Discussion	Yes
Funding	22	Funding source and role given — Acknowledgements	Yes
